# Vulgar Habits

**Published:** 1883-01

**Authors:** 


VULGAR HABITS.—Asking questions private and personal is a vulgar habit, and telling your own business, which no one wants to hear, is another. Asking the cost of a present that has been made to you, loud talking in public, hard staring at table, insolent disrespect to husband, wife, sister or brother, showing temper in trifles, and making scenes in public, showing an embarrassing amount of fondness, and making love in public, covert sneers, of which people can see the *animus*, if they do not always understand the drift; persistent egotism, which talks forever of itself, and cannot even feign the most passing interest in another, detraction of friends, and it may be of relatives, a husband telling of his unpleasantnesses, a wife complaining of her husband's faults, the bold assumption of superiority, and the servile confession of infinite unworthiness—all these are signs and evidences of vulgarity—vulgarity of a far worse type than that which eats its fish with a steel knife, and says "You was," and "Each of the men were."

